# Separation From the Life Partner and Exit From Self-Employment

**DOI:** 10.3389/fpsyg.2020.01118

**Published:** 2020-07-23

**Authors:** Leanne van Loon, Jolanda Hessels, Cornelius A. Rietveld, Peter van der Zwan

**Affiliations:** ^1^Department of Applied Economics, Erasmus School of Economics, Erasmus University Rotterdam, Rotterdam, Netherlands; ^2^Erasmus University Rotterdam Institute for Behavior and Biology, Erasmus University Rotterdam, Rotterdam, Netherlands; ^3^Department of Business Studies, Leiden Law School, Leiden University, Leiden, Netherlands

**Keywords:** exit, life partner, self-employment, social functioning, household income

## Abstract

The survival of businesses in the market often hinges on contributions of the business owner’s household members. Partners of the self-employed as well as their children may, for example, provide emotional support but also cheap and flexible labor. Although the household composition of self-employed individuals has been analyzed in many earlier studies, little is known about what happens to the self-employed individual and his or her business when one separates from a life partner. We argue that separation from a life partner has profound financial and social consequences for the business owner. Specifically, we propose that a decrease in household income and social functioning (which is the degree of interference with social activities due to mental and/or physical problems) after separation from the life partner may lead to an exit from self-employment. Our empirical analysis draws on data from the longitudinal HILDA (Household, Income and Labour Dynamics in Australia) survey, for the period 2002–2017. Based on information from 4,044 self-employed individuals aged 18–64 years (18,053 individual-year observations), we find that separating from the life partner in the past year significantly increases the probability of exit from self-employment in the next year. Furthermore, we find that the positive association between separation from the life partner and exit from self-employment can be explained for 29.7% by a reduction in social functioning and for 10.7% by a reduction in household income. We study five exit routes out of self-employment and find that separation from the life partner mainly increases the probabilities of becoming a wage worker and of re-entering self-employment after experiencing an exit. For exit to unemployment or to a position outside the labor force (voluntarily inactive/retirement or any other non-labor force position), we find insignificant relationships with separation from the life partner. Furthermore, for all exit routes except retirement, we find significant indirect effects implying that decreased household income and levels of social functioning are important mechanisms through which separation from the life partner is related to exit from self-employment.

## Introduction

Individuals from all countries and cultures commonly aspire sharing a lifelong dedicated relationship with an intimate partner (Halford and Snyder, 2012). Yet, such relationships do not always work out well and may end in a separation. A separation refers to a situation in which two life partners (i.e., a married or non-married couple) decide or arrange to stop living or being together as a couple.^1^ Many studies have focused on the financial and social consequences for individuals after a separation from a life partner such as through divorce (Holmes and Rahe, 1967; Pai and Carr, 2010). This topic has gained more attention partly due to the significant increase in divorce rates over the past centuries. According to Eurostat (2019), the divorce rate in the European Union increased over the period 1965–2016, while the marriage rate decreased during the same period. A similar trend can be seen in the United States in the past century, although from 2000 onward, the divorce rate has declined somewhat due to millennials being pickier and marrying at an older age (Cohen, 2019). In Australia, the number of divorces per 1,000 Australian residents rose in the 1960s and 1970s and peaked at 4.6 after the introduction of the Family Law Act 1975. Thereafter, the divorce rate steadily decreased to 2.0 in 2017 (Australian Institute of Family Studies, 2020).

A separation or divorce is, in many cases, a negative and stressful event. Studies have identified the loss of emotional support, economic decline, and health problems as negative consequences of a separation (Amato, 2000; Poortman, 2000; McManus and DiPrete, 2001). While being married is positively associated with work effectiveness and work performance (Selmer and Lauring, 2011), divorces lead to a deterioration of living conditions, which might indirectly result in a reduction of an individual’s performance at work (Hetherington et al., 1976). Interestingly, it has not been thoroughly studied whether and how the separation from a partner influences the performance of self-employed individuals. However, the self-employed tend to operate in highly uncertain business environments, and it is known that (*de facto*) relationships (such as marriage) offer them stability. Given the importance of the presence of a life partner for self-employed individuals, separations can be expected to have an impact on the self-employed’s business. The self-employed represent an important and vital part of today’s labor force (Van Stel and van der Zwan, 2019). Therefore, in light of the current demographic trend sketched above, studying the consequences of separation from the life partner for the self-employed and their businesses is highly relevant.

In this paper, we focus on whether the separation from a life partner is related to an exit from self-employment. Exit from self-employment is seen as an important process for business owners, as well as an important event for the economy, the industry, and related firms (DeTienne, 2010). Exit from self-employment can be defined as “the process by which the founders of privately held firms leave the firm they helped to create; thereby removing themselves, in varying degree, from the primary ownership and decision-making structure of the firm” (DeTienne, 2010, p. 203). One may categorize exits from self-employment in several ways such as in terms of sale or liquidation (Wennberg et al., 2010), the (in)voluntary character of the exit (Coad, 2014), or what happens after an exit. The self-employed individual may end up in another labor market position after an exit (wage work and unemployment), (s)he may decide to set up a new business (becoming self-employed again) or may end up outside the labor force such as in retirement (Hessels et al., 2018). A growing body of literature recognizes the importance of exit from self-employment and studies this phenomenon to gain insights into what causes an exit (Wennberg et al., 2010).

Given the nature of a separation, its consequences are likely to be radical but also diverse. We investigate the roles of income and social functioning as mechanisms through which separation is related to an exit from self-employment. One of the main consequences frequently mentioned by individuals who separated from a life partner are the implications for one’s financial situation such as through reduced household income (Poortman, 2000; McManus and DiPrete, 2001; Sevak et al., 2003; Andreß et al., 2006). Since one’s financial situation may impact the possibility and decision to remain in self-employment with one’s current business (Bird and Wennberg, 2016), we suspect that a separation may lead to an exit from self-employment because of a decline in household income.

Another main implication of separation is its social effect. Women who separated from their life partner through a divorce, for example, often mention to have problems with socialization and problems with feelings of failure as well as to experience feelings of shame (Bloom et al., 1978). Also, men who separated from their life partner through a divorce indicated experiencing emotional problems due to feelings of loneliness (Bloom et al., 1978). Furthermore, they felt that they were functioning less in both social and work situations (Hetherington et al., 1976). Perhaps even the most important social consequence for both men and women is the loss of emotional support from the partner (Amato, 2000). Based on these findings, we suspect that an individual’s social functioning is affected negatively after a separation from a life partner. Reduced social functioning is likely to negatively affect the self-employed and their businesses. Having less social abilities tends to lead to poorer decision making (Bar-On et al., 2004). Similarly, having better social skills and a higher mental ability is found to be associated with higher salary levels (Ferris et al., 2001). Therefore, reduced social functioning as a consequence of separation from a life partner might be detrimental for the self-employed and eventually lead to an exit from self-employment.

The added value of this paper is at least twofold. First, this paper contributes to the growing body of literature on the event of an exit from self-employment or entrepreneurship. Such exits are found to have significant consequences for several actors. For example, there may be psychological consequences for the self-employed individual, cash-flows into the firm, competitive effects for the industry, and a redistribution of wealth in the (regional) economy (DeTienne, 2010). When taking the example of a divorce of a self-employed individual who is married in community of property, the divorce might result in a forced liquidation of the self-employed’s business with subsequently the possible negative consequences for the self-employed individual, firm, industry, and economy. From a welfare economic point of view, the liquidation of the firm may be suboptimal for overall welfare. Gaining insights into the mechanisms between separation from the life partner and exit from self-employment may help to develop policies to counter negative welfare effects.

Second, this paper contributes to the literature on the economic consequences of a separation from a life partner. While many studies focus on the mental and physical consequences of the loss of a partner, the consequences of separating from a life partner for a self-employed individual have not yet been considered. Given the severe mental and physical consequences of a separation from a life partner (Menaghan and Lieberman, 1986; Lorenz et al., 2006), it is a valuable addition to study the possible subsequent economic consequences. With that, we move past the point of studying only the direct consequences of losing a partner and introduce a new direction for research focusing on the more down-stream consequences of a separation from the partner.

In the next sections, we review the relevant literature, and we formulate our hypotheses. We test these hypotheses using longitudinal data from the Household, Income and Labour Dynamics in Australia (HILDA) survey (Summerfield et al., 2019). In Australia, 9.6% of those active in the labor market were self-employed in 2018 (Organisation for Economic Co-operation and Development, 2020), and this percentage is relatively low compared to other OECD countries. However, according to the Global Entrepreneurship Monitor, the number of individuals actively engaged in starting and running new businesses is above the average of developed countries and similar to levels in the United States (Steffens and Omarova, 2019). The Global Entrepreneurship Monitor also indicates that many exits from self-employment in Australia are not failures, but rather represent successful business exits or better opportunities for the (formerly) self-employed individual. The results of our analysis are presented in the Results section, and they indicate that a separation from a life partner increases the probability of an exit from self-employment in the next year. This relationship can, for a substantive and significant part, be explained by a reduction in social functioning and, to a smaller extent, by a reduced household income. Results of additional (robustness) analyses are reported in the Section “Subsample Analyses and Robustness Check.” In the Section “Discussion and Conclusion,” we discuss the findings of our study as well as their implications and propose directions for future research.

## Theory and Hypotheses

### The Presence of a Life Partner and Self-Employment

Someone’s family context is increasingly considered to be an important factor influencing someone’s employment status, including being and remaining self-employed (Sanders and Nee, 1996; Bird and Wennberg, 2016). The role of the partner for becoming self-employed as well as the influence of a partner on the performance of their partner’s business has become clearer over the past decades. Özcan (2011) found that the relationship context ultimately shapes the constraints and resources as well as the motivations of men and women to choose self-employment. Marriage is an important determinant of transitioning into self-employment (Simoes et al., 2016), and one of the main resources underneath this strong tie is the additional social capital that can be accessed through marriage. Sanders and Nee (1996) studied immigrant entrepreneurs and found that many immigrants indicated having a chronic shortage of capital. However, it was found that immigrants often have strong family ties that provide them with financial resources and enables the pooling of labor. Important sources of financial and social capital when starting a business were their family and extended family. Having a partner provides additional resources through his or her family. Aldrich and Cliff (2003) go even further and suggest that family and marriage positively influence the recognition of opportunities, decisions to launch a business or product, the mobilization of resources, and the implementation of strategies, processes, and structures. They also mention the enlarged family labor pool through stepfamilies that come with divorces and remarriages.

Besides the transition into self-employment, the partner is also found to be an important driver behind the success of the self-employed. Bratkovic et al. (2009) studied the role of the female partner for the self-employed male, and they conclude that she plays a crucial role in the resource-information acquisition process of the firm of her husband. By being active in the network of the firm, she is able to gather valuable information for the business as well as to maintain valuable contacts with the network. Furthermore, besides being of value for the business, the partner also provides emotional support for the self-employed individual, which is found to be positively related with performance of the business (Bosma et al., 2004). Finally, marriage or a registered partnership can also be seen as a condition that offers stability for the self-employed individual while operating in a risky and uncertain business environment (Brown et al., 2006). Having an employed partner with a stable income offers the possibility of spreading risks within the family or household. This is a condition unavailable to someone without a life partner (Henley, 2007).

### Financial and Social Consequences of a Separation

A separation from a life partner such as through a divorce can be seen as a stressful event followed by both social and psychological distress (Miller et al., 1998). Given the severe nature of the event, a separation from the life partner is found to have some drastic consequences. Based on the literature, we distinguish two consequences of a separation that we expect to be of relevance for a self-employed individual. The first consequence refers to the adverse financial consequences or a reduction in income, and the second consequence concerns the reduction in social functioning.

First, a separation from the partner is found to have severe economic consequences, especially for women. Andreß et al. (2006), for example, found—based on data for Belgium, Germany, Great Britain, Italy, and Sweden—that household income is negatively affected by separations for both sexes but particularly for women. Morgan (1989) looked into United States women who separated from their life partner through a divorce and found that during the first 5 years after the terminated marriage, 25% of these women experienced a period of poverty. However, it was also noted that there was considerable movement in and out of poverty, suggesting that the economic decline was not necessarily a long-term condition. Another economic consequence following a separation or divorce is the division of assets between the former life partners. Weitzman (1980) found that over the period 1968–1977 in the United States, the majority of businesses were awarded to the husband. However, this was also in a period that an exact division of assets was not required under the law. Nowadays, it can be expected that in some cases, a marital dissolution might also lead to a dissolution of the business owned by both the former life partners.

Second, a separation can have social implications and result in a reduction in social functioning of the individual. Social functioning refers to both the extent that the respondent experiences negative interferences with social activities due to physical and/or mental problems and the total social time available (Ware, 2000). Thus, the reduction in social functioning stems from physical and psychological problems that often come with a separation. Regarding physical problems, it is well known that a separation from the life partner is associated with a deterioration of physical health. Especially, the situation of experiencing chronic stress due to a separation is related to having more health problems (Lorenz et al., 2006; Hughes and Waite, 2009). It is also found that individuals who spend more time being divorced, without remarrying, show more chronic conditions and more mobility limitations than individuals with a continuing marriage (Hughes and Waite, 2009). Furthermore, Williams and Umberson (2004) found that divorced men and women, compared to married individuals, have a poorer self-assessed health. Regarding the mental or psychological consequences of a separation, studies have found that individuals who separated from their life partner through a divorce are significantly more depressed 4 years after the divorce than their married counterparts (Menaghan and Lieberman, 1986). These psychological consequences are related to social aspects. One of the main factors found to increase the deterioration of mental health is the loss of emotional support from the life partner after a separation (Amato, 2000). Such emotional support can be seen as social capital (Bosma et al., 2004). In sum, the physical and mental problems after a separation will likely negatively interfere with engagement in social activities and, hence, result in reduced social functioning. Research, indeed, indicates that men who separated from their life partner through a divorce feel that they are functioning less in social situations (Hetherington et al., 1976) and that divorced women often have problems with socialization (Bloom et al., 1978). Besides the negative internal social consequences for the individual, the individual often also loses access to the social network and family of his or her former partner after a separation. Given that it was found that the wife of a self-employed individual plays a crucial role by gathering valuable information by maintaining the network (Bratkovic et al., 2009), a self-employed individual will likely lose valuable resources when separating from his or her partner.

### Separation and Exit From Self-Employment

An exit from self-employment is not only an impactful event for self-employed individuals; it also has implications for the economy, the industry, and related firms, for example, through the resources that are released through an exit (DeTienne, 2010). Recent literature has focused on the reasons for an exit from self-employment or entrepreneurship. Factors contributing to such an exit can be found at the micro-level. For example, research suggests that an individual’s mental health (Hessels et al., 2018), work and leisure satisfaction (Van der Zwan et al., 2018), and initial work experience and capital (Taylor, 1999) may affect exit decisions. Macro-economic conditions may also play a role for decisions to exit from self-employment or entrepreneurship, such as the business cycle (Everett and Watson, 1998; Koellinger and Roy Thurik, 2012) and competition within industries (Dunne et al., 1988). Even though many reasons for exit from self-employment have been studied, the impact of family related factors is currently underexposed.

Some hints for a possible association between the separation from a life partner and an exit from self-employment can, nevertheless, be found in the recent literature. Wennberg et al. (2010) distinguish four exit routes, including “harvest liquidation,” which refers to a situation in which a high-performing firm is sold. As an example of why someone would choose this exit route, they mention a divorce (see also Coad, 2014). When partners both own the business and they come to a marital dissolution, in most cases, they will have to distribute their assets. This might lead to a forced sale of the business even for firms that perform well. Also, Ronstadt (1986) found that family related problems might lead to an exit from self-employment. In a survey among 95 ex-self-employed individuals, 21% of the respondents indicated that the reason for their exit was due to financial problems and personal/family problems. Another 11% indicated that their exit was due to personal and/or family problems alone. Divorces are included in this category. These findings suggest that family and personal problems, which might include a separation or a divorce from a life partner, constitute an important reason for an exit from self-employment. Finally, in an exploratory study Galbraith (2003) found some linkages between marital status and an exit from self-employment. He recognizes marital dissolution as having a negative impact on the short-term performance of especially small businesses.

### Hypotheses

To summarize, prior research indicates that having a life partner positively influences the probability of becoming self-employed (Özcan, 2011) as well as the performance of the self-employed person (Bosma et al., 2004). For example, a life partner gives access to crucial resource information through maintaining a social network (Bratkovic et al., 2009). When separated from the partner, the self-employed individual loses valuable resources as well as emotional support. This leads us to hypothesis 1.

**Hypothesis 1:** *There is a positive relationship between separation from the life partner and an exit from self-employment*.

One of the main negative consequences of a separation is the reduction of household income. Such a reduction in income could make it more likely for self-employed individuals to exit from self-employment. Therefore, we hypothesize:

**Hypothesis 2:** *Household income mediates the positive relationship between separating from the life partner and an exit from self-employment.*

A second main consequence of a separation is the negative impact on one’s social functioning through physical and psychological effects. Importantly, especially social capital is found to be important for the success of a self-employed individual (Stam et al., 2014). Given the expected decrease in social functioning of the self-employed individual after separating from a partner, we hypothesize:

**Hypothesis 3:** *Social functioning mediates the positive relationship between separating from the life partner and an exit from self-employment.*

## Data and Methods

### Data and Sample

Our empirical analysis relies on longitudinal data from the Household, Income and Labour Dynamics in Australia (HILDA) survey. This panel dataset contains, among others, information on work-related characteristics and family characteristics. We use annual data for the period 2002–2017.^2^ Hence, individuals are followed for a maximum period of 16 years in total in our sample. Our analysis sample consists of individuals who have been self-employed in at least one annual wave in the period 2002–2017. We restrict our sample to individuals aged 18–64. The upper bound of 64 years is chosen because Australia’s eligibility age for age pension is 65 years. The estimation sample amounts to 18,053 person-year observations (4,044 individuals).

### Variables

#### Dependent Variable: Exit From Self-Employment

The main dependent variable is a binary variable indicating whether an individual has exited self-employment between time *t* and *t* + 1, indicated as 1, or is still in self-employment at time *t* + 1, indicated as 0.

Furthermore, we distinguish among several “exit routes.” Given the survey’s focus on the individual rather than the business, we focus on exit from self-employment (individual exit) rather than business exit, and hence, the exit routes inform us about an individual’s employment at time *t* + 1 after experiencing an exit from self-employment between *t* and *t* + 1. We distinguish the following five routes the self-employed individual could follow after an exit. First of all, the individual could exit to wage work. The second route consists of exit to unemployment. The third route is exit toward a position outside of the labor force (this includes home duties, childcare, an unpaid voluntary job, traveling, illness, etc.). The fourth route refers to a specific position outside the labor force, i.e., “retirement or voluntarily inactive.” It is important to distinguish this specific position outside the labor force because it is explicitly a voluntary one. Previous research has associated this position with relatively successful exits in the context of business exit (Coad, 2014), and its occurrence is relatively frequent. The fifth and final route refers to individuals leaving self-employment and becoming self-employed again (so-called serial self-employment) (Parker, 2013). All in all, we generate a categorical exit variable with these five exit routes, while the value of 0 refers to individuals who are still in self-employment at time *t* + 1.

#### Independent Variable: Separation From the Life Partner (Life Event)

The main independent variable reflects whether someone indicated to have been separated from their life partner between time *t* − 1 and time *t*. By measuring exit in the subsequent period (between time *t* and *t* + 1), we reduce potential bias due to reverse causality. The questionnaire consists of a list of life events and a respondent had to tick the box if this life event was applicable to him or her. One of the life events was “Separated from spouse or long-term partner.” The main advantage of measuring the separation through the life event question over constructing a variable based on an individual’s (registered) marital status—see also the robustness check in the “Subsample Analyses” section—is that it also includes information about non-registered marriages, such as *de facto* relationships. In addition, only one wave is needed to extract information rather than two consecutive years in case of the (official) marital status variable, leading to a larger sample size.

#### Mediator: Household Income

Total disposable household income over the past year measured at time *t* is taken as our income measure (logarithmically transformed). Extensive information about the construction of this income measure is provided in Summerfield et al. (2019).

#### Mediator: Social Functioning

The variable indicating *social functioning* is constructed based on the Short Form Health Survey (SF-36) and consists of two components: social extent and social time (Ware, 2000). The variable measures, at time *t*, to what extent the respondent experiences negative interference with social activities due to mental and/or physical problems. Specifically, the relevant items are as follows: (1) During the past 4 weeks, to what extent has your physical health or emotional problems interfered with your normal social activities with family, friends, neighbors, or groups? (answer possibilities: not at all, slightly, moderately, quite a bit, extremely); and (2) During the past 4 weeks, how much of the time has your physical health or emotional problems interfered with your social activities (like visiting with friends, relatives, etc.)? (answer possibilities: all, most, some, a little, and none of the time). The answers have been transformed into a score on a 0–100 scale (Ware et al., 2000). A higher score represents better social functioning.

#### Control Variables

Based on previous research, a broad set of control variables is included in the empirical analysis (Patel and Thatcher, 2014; Parker, 2018). The individual-specific control variables, at time *t*, consist of gender (0 = female; 1 = male), age (18–64), age squared, education (based on the total number of years of schooling) (Leigh and Ryan, 2005), the number of own resident children, and the state of residence (dummy variables for the Australian states). Furthermore, some job-specific control variables were added including the number of working hours per week (in logarithms), the duration of current employment in years (in logarithms)^3^, and industry of employment^4^. Finally, we control for the year of the interview.

### Methods

Given the nature of our dependent variable, discrete-time survival models are used. Allison (1982) already showed that such survival models can be operationalized by applying regression models for binary dependent variables (in the case of distinguishing between exit and survival) and multinomial logistic models (in the case of our exit routes). In other words, we perform binary and multinomial logistic regressions to fit discrete-time logistic hazard models. These models can include time-varying variables and right-censored observations, both present in our case. Examples of right-censored observations are individuals who are still self-employed in 2017. We do not use clustered standard errors (Allison, 1982). Note that the hazard rate—the probability that an exit occurs at time *t* given that it has not occurred until time *t*—is assumed to be different for each of the 16 years under investigation given the inclusion of wave dummies in the specification.

First, we perform a binary logistic regression with the variable indicating whether an exit from self-employment occurs between time *t* and *t* + 1 as the dependent variable. The independent variable reflects whether someone experienced a separation from a life partner between time *t* − 1 and *t*. Second, a multinomial logit regression is performed with a categorical variable indicating the various exit routes as the dependent variable.^5^ The reference category in the multinomial logit regression is survival (still in self-employment at time *t* + 1) such that the coefficients belonging to the five exit routes can be interpreted relative to staying in self-employment. Third, household income and social functioning, measured at time *t*, are added to our models as possible mediators. Given the non-linear nature of our regression models (binary and multinomial logistic), we assess the magnitude of the possible mediating (indirect) effects using the KHB method (Karlson et al., 2012). In sum, exit can take place between time *t* and *t* + 1, separation between time *t* − 1 and *t*, and the mediators and all control variables are measured at time *t*. Working with larger lags of variables would reduce the estimation sample, which is not preferable given the already relatively small numbers of exit and separation instances.

A few additional analyses were performed to see whether the main results also hold for subgroups of individuals. We perform separate analyses for self-employed individuals without employees and those with employees. Also, we report the results of subsample analyses based on gender (men versus women), age (younger versus older individuals), education (higher versus lower educated individuals), the duration of the marriage (shorter versus longer relationships), the presence of children in the household just before separation (no children present versus children present), and living area (rural versus urban).

Two robustness checks were performed. First, we base the independent variable on the self-employed’s marital status as revealed in the questionnaire (rather than using the question on the life events). Second, we control for the fact that certain factors influence the decision to separate, such as socio-demographic characteristics but also previous values of income and social functioning. We therefore present the results of a propensity score matching exercise (Sbarra et al., 2014) in which each observation corresponding to a separation event is matched with an observation corresponding to non-separation based on similar propensity scores.^6^ Both observations then have a similar profile in terms of the covariates predicting the propensity score, i.e., the socio-demographic variables, job characteristics, and lagged values of income and social functioning.

## Results

Table 1 shows the descriptive statistics of the dependent variables, our independent variable, the mediating variables, and the control variables. In total, there are 18,053 individual-year observations from 4,044 distinct individuals. Importantly, 16.7% of the person-year observations constitute an exit from self-employment. Exit to wage work is the most prevalent exit route. Furthermore, the prevalence of separation in the sample is 3.3%. Table 2 shows the correlation table.

**TABLE 1:** Descriptive statistics analysis sample.

Variable	Mean	*SD*	Minimum	Maximum
Exit	0.17	0.37	0	1
Exit to wage work	0.10	0.30	0	1
Exit to unemployment	0.01	0.09	0	1
Exit to non-labor force	0.03	0.17	0	1
Exit to voluntarily inactive	0.01	0.11	0	1
Exit to new self-employment	0.01	0.11	0	1
Separation	0.03	0.18	0	1
Social functioning	86.76	19.76	0	100
Household income (log)	11.27	0.76	4.25	13.74
Male	0.64	0.48	0	1
Age	44.90	10.94	18	64
Education	12.57	2.11	8	17
Children	1.10	1.21	0	5
Hours worked (log)	3.52	0.69	0.01	4.94
Work tenure (log)	1.74	1.13	0	3.93

*Table based on 18,053 individual-year observations (from 4,044 individuals). SD, standard deviation. Descriptive statistics for state of residence, sector, and year of the survey are available upon request from the authors.*

**TABLE 2:** Correlation table analysis sample.

	1	2	3	4	5	6	7	8	9	10
(1) Exit	1.00									
(2) Separation	0.03	1.00								
(3) Social functioning	–0.08	–0.12	1.00							
(4) Household income (log)	–0.06	–0.10	0.13	1.00						
(5) Male	–0.08	0.01	0.05	–0.01	1.00					
(6) Age	–0.06	–0.07	0.00	0.04	0.05	1.00				
(7) Education	–0.01	–0.02	0.03	0.24	–0.05	–0.04	1.00			
(8) Children	–0.04	–0.08	0.04	0.20	–0.04	–0.11	0.04	1.00		
(9) Hours worked (log)	–0.19	0.00	0.07	0.03	0.39	–0.01	–0.03	–0.02	1.00	
(10) Work tenure (log)	–0.15	–0.06	0.05	0.09	0.11	0.45	–0.10	0.04	0.11	1.00

*Table based on 18,053 individual-year observations (from 4,044 individuals). Pearson correlations for the exit routes, state of residence, sector, and year of the survey are available upon request from the authors.*

In Table 3 (panel I), the results of a binary logistic regression explaining exit from self-employment in the next period (without distinguishing between different exit routes) are presented. The results reveal that separating from the partner is significantly and positively related with an exit from self-employment. This result confirms Hypothesis 1. Further analyses, displayed in panel II of Table 3, show that, opposed to remaining in self-employment, separation from the life partner is associated with higher probabilities of exiting toward wage work and of becoming self-employed again. There is no significant relation between separation from the life partner and an exit toward unemployment or a position outside of the labor force (either voluntarily inactive/retirement or any of the other non-labor force possibilities). Consequently, it can be concluded that a separation from the life partner increases the probability of experiencing an exit from self-employment, and that the most likely exit routes are exits toward paid employment and becoming self-employed again (versus survival). It is relatively unlikely for a separated self-employed person to be jobless after experiencing an exit from self-employment.

**TABLE 3:** Results of binary and multinomial logistic regressions with *Exit from self-employment in the next period* as the dependent variable.

	Binary logit (I)		Multinomial logit (II)									
	Exit		Exit to wage work		Exit to unemployment		Exit to non-labor force		Exit to voluntarily inactive		Becoming self-employed again	
	Coeff.	*SE*	Coeff.	*SE*	Coeff.	*SE*	Coeff.	*SE*	Coeff.	*SE*	Coeff.	*SE*
Separation	0.286***	0.103	0.253**	0.123	0.394	0.357	0.240	0.225	0.205	0.479	0.622**	0.270
Male	−0.102**	0.049	−0.126**	0.060	0.474**	0.194	−0.586***	0.105	0.191	0.163	0.416**	0.166
Age/10	−0.702***	0.147	–0.271	0.182	–0.171	0.555	–0.323	0.313	−1.576*	0.825	–0.075	0.487
Age/10 squared	0.080***	0.017	0.015	0.021	0.028	0.065	0.035	0.037	0.317***	0.083	–0.003	0.058
Education	–0.014	0.011	0.006	0.014	–0.016	0.045	−0.082***	0.024	−0.078**	0.035	0.043	0.037
Children	−0.041**	0.020	−0.047*	0.024	–0.068	0.081	–0.009	0.043	−0.235**	0.102	0.028	0.062
Hours worked	−0.540***	0.029	−0.304***	0.038	−0.697***	0.010	−0.917***	0.049	−0.984***	0.075	–0.104	0.114
Work tenure	−0.310***	0.021	−0.299***	0.026	−0.644***	0.086	−0.280***	0.046	–0.102	0.067	−0.527***	0.069
Observations	18,053		18,053									
Pseudo *R*^2^	0.06		0.09									

***p ≤ 0.01, **p ≤ 0.05, *p ≤ 0.10 (two sided). Coeff., coefficient; SE, standard error. The estimates corresponding to state of residence, sector, and year of the survey are available upon request from the authors. Reference category in multinomial logit regression: staying in self-employment.

In Table 4, the results of the same binary and multinomial logistic regressions are presented as in Table 3, but here, the mediators, *household income* and *social functioning*, at time *t* are included in the model. The results in panel I of Table 4 reveal that the relationship between the separation from the partner and the probability of an exit from self-employment is mediated by both *household income* and *social functioning*. That is, the indirect effects of both variables are significant and positive. *Social functioning* is the most important mediator, explaining 29.7% of the relationship between separation and exit, while the indirect effect corresponding to *household income* explains 10.7% of the total effect. Hence, we find that decreased levels of household income and social functioning are important mechanisms through which separation is related to an exit from self-employment.^7^ In conclusion, we find support for both Hypothesis 2 and Hypothesis 3.

**TABLE 4:** Results of binary and multinomial logistic regressions with *Exit from self-employment in the next period* as the dependent variable.

	Binary logit (I)		Multinomial logit (II)									
	Exit		Exit to wage work		Exit to unemployment		Exit to non-labor force		Exit to voluntarily inactive		Becoming self-employed again	
	Coeff.	*SE*	Coeff.	*SE*	Coeff.	*SE*	Coeff.	*SE*	Coeff.	*SE*	Coeff.	*SE*
Separation	0.169	0.105	0.190	0.125	0.171	0.363	–0.098	0.230	0.232	0.483	0.514*	0.275
Household income	−0.102***	0.030	–0.060	0.037	−0.503***	0.090	−0.257***	0.061	0.089	0.094	–0.017	0.100
Social functioning	−0.007***	0.001	−0.004***	0.001	–0.006	0.004	−0.018***	0.002	0.001	0.003	−0.008**	0.003
Male	−0.100**	0.049	−0.125**	0.060	0.442**	0.193	−0.607***	0.105	0.185	0.163	0.420**	0.166
Age/10	−0.731***	0.147	–0.282	0.182	–0.264	0.056	–0.436	0.313	−1.560*	0.822	–0.087	0.486
Age/10 squared	0.083***	0.017	0.016	0.021	0.040	0.065	0.046	0.037	0.315***	0.082	–0.002	0.058
Education	–0.007	0.011	0.010	0.014	0.014	0.045	−0.065***	0.025	−0.082**	0.036	0.045	0.037
Children	–0.022	0.020	–0.037	0.025	0.019	0.082	0.048	0.043	−0.253**	0.104	0.035	0.063
Hours worked	−0.526***	0.029	−0.297***	0.038	−0.656***	0.010	−0.886***	0.049	−0.976***	0.076	–0.095	0.114
Work tenure	−0.301***	0.021	−0.294***	0.026	−0.615***	0.087	−0.254***	0.046	–0.105	0.068	−0.521***	0.070
*Indirect effects*												
Household income	0.030***	0.009	0.018	0.011	0.150***	0.031	0.077***	0.020	–0.026	0.028	0.005	0.030
Social functioning	0.084***	0.013	0.046***	0.016	0.073	0.047	0.223***	0.026	–0.009	0.043	0.095**	0.039
Total indirect effect	0.115***	0.016	0.064***	0.019	0.224***	0.054	0.299***	0.032	–0.035	0.049	0.100**	0.047
Observations	18,053		18,053									
Pseudo *R*^2^	0.07		0.10									

Regressions include the mediators social functioning and household income. ***p ≤ 0.01, **p ≤ 0.05, *p ≤ 0.10 (two sided). Coeff., coefficient; SE, standard error. The estimates corresponding to state of residence, sector, and year of the survey are available upon request from the authors. Reference category in multinomial logit regression: staying in self-employment.

Panel II of Table 4 shows the results for the multinomial logit model. We observe that the coefficient of the separation variable is no longer significant for exit to wage work (*p* = 0.13) and significant at the 10% level for exit to new self-employment (*p* = 0.06) while controlling for household income and social functioning. We find significant and positive total indirect effects for all exit routes except for voluntarily inactive/retirement. In general, we find larger indirect effects for social functioning than for household income, and the large indirect effect for social functioning for the non-labor force route stands out. The fact that the indirect effects for the voluntarily inactive route are non-significant is not surprising given that the coefficients of household income and social functioning are not significant in Table 4 for this exit route.

## Subsample Analyses and Robustness Checks

### Subsample Analyses

Tables 5, 6 show results from binary logit regressions for subsamples of individuals based on the number of employees, gender, age, education, duration of marriage, children before separation, and urbanization. The regressions results inform us about the relationship between separation and exit from self-employment without considering the mediators. For completeness, we also report on the indirect effects at the bottom of the tables once the mediators are added to the models. The complete set of regression results with the mediators included is available upon request from the authors.

**TABLE 5:** Results of binary logistic regressions with *Exit from self-employment in the next period* as the dependent variable.

	Self-employed individuals without employees		Self-employed individuals with employees		Self-employed women		Self-employed men		Self-employed individuals with age <45		Self-employed individuals with age ≥45		Self-employed individuals with low education		Self-employed individuals with high education	
	Coeff.	*SE*	Coeff.	*SE*	Coeff.	*SE*	Coeff.	*SE*	Coeff.	*SE*	Coeff.	*SE*	Coeff.	*SE*	Coeff.	*SE*
Separation	0.285**	0.119	0.131	0.236	0.323*	0.167	0.245*	0.133	0.299**	0.128	0.212	0.180	0.388***	0.114	–0.134	0.253
Male	–0.079	0.062	−0.287***	0.091					−0.157**	0.070	–0.063	0.069	−0.139**	0.058	0.019	0.091
Age/10	−0.637***	0.173	−0.657**	0.310	−0.469*	0.239	−0.822***	0.189	–0.102	0.427	−3.385***	1.100	−0.869***	0.164	–0.066	0.353
Age/10 squared	0.071***	0.020	0.076**	0.036	0.046	0.028	0.097***	0.022	0.002	0.064	0.331***	0.101	0.101***	0.019	0.000	0.040
Education	–0.023	0.014	0.020	0.021	−0.035**	0.016	0.002	0.016	−0.035**	0.017	0.001	0.015	–0.020	0.025	0.068	0.052
Children	–0.012	0.026	–0.049	0.037	–0.031	0.032	−0.058**	0.026	−0.078***	0.028	–0.020	0.030	–0.032	0.024	−0.070*	0.039
Hours worked	−0.532***	0.035	−0.468***	0.061	−0.492***	0.038	−0.589***	0.048	−0.565***	0.043	−0.532***	0.040	−0.511***	0.034	−0.649***	0.056
Work tenure	−0.299***	0.026	−0.283***	0.041	−0.259***	0.034	−0.334***	0.027	−0.377***	0.033	−0.259***	0.028	−0.321***	0.024	−0.271***	0.043
Observations	10,210		7,014		6,487		11,566		8,471		9,582		13,115		4,938	
Pseudo *R*^2^	0.06		0.06		0.06		0.06		0.07		0.06		0.06		0.07	

***Indirect effects based on regressions with mediators included***																

Household income	0.020**	0.010	0.033	0.021	0.044**	0.018	0.024**	0.011	0.036***	0.013	0.032**	0.015	0.037***	0.012	0.020	0.015
Social functioning	0.086***	0.017	0.058***	0.021	0.079***	0.020	0.091***	0.018	0.100***	0.020	0.066***	0.018	0.092***	0.016	0.053**	0.024
Total ind. effect	0.106***	0.019	0.091***	0.028	0.123***	0.026	0.114***	0.020	0.136***	0.023	0.098***	0.022	0.128***	0.019	0.073***	0.027

Subsample analyses for self-employed individuals without and with employees, for self-employed women and men, for younger (age < 45) and older (age ≥ 45), and for lower educated (<12.5 years of schooling) and higher educated (≥ 12.5 years) self-employed individuals. ***p ≤ 0.01, **p ≤ 0.05, *p ≤ 0.10 (two sided). Coeff., coefficient; SE, standard error. The number of employees is not known for 829 person-year observations. The estimates corresponding to state of residence, sector, and year of the survey are available upon request from the authors.

**TABLE 6:** Results of binary logistic regressions with *Exit from self-employment in the next period* as the dependent variable.

	Self-employed individuals in a short relationship		Self-employed individuals in a long relationship		Self-employed individuals without children		Self-employed individuals with children		Self-employed individuals living in rural areas		Self-employed individuals living in urban areas	
	Coeff.	*SE*	Coeff.	*SE*	Coeff.	*SE*	Coeff.	*SE*	Coeff.	*SE*	Coeff.	*SE*
Separation	0.343**	0.170	0.743***	0.289	0.299**	0.146	0.322*	0.170	0.353**	0.174	0.241*	0.129
Male	−0.181**	0.079	–0.004	0.090	–0.084	0.071	−0.127*	0.074	–0.074	0.082	−0.113*	0.061
Age/10	−1.029***	0.268	−2.339***	0.821	−0.577***	0.185	−1.261***	0.302	−0.760***	0.246	−0.658***	0.185
Age/10 squared	0.120***	0.032	0.245***	0.078	0.062***	0.022	0.148***	0.034	0.083***	0.028	0.076***	0.022
Education	–0.010	0.018	–0.004	0.019	–0.019	0.017	–0.011	0.016	−0.043**	0.020	0.00002	0.014
Children	–0.038	0.031	0.033	0.039	–0.004	0.112	0.016	0.033	–0.051	0.033	–0.036	0.025
Hours worked	−0.543***	0.048	−0.610***	0.050	−0.556***	0.043	−0.556***	0.043	−0.484***	0.048	−0.580***	0.037
Work tenure	−0.322***	0.035	−0.264***	0.036	−0.266***	0.031	−0.336***	0.031	−0.259***	0.034	−0.332***	0.027
Observations	7,545		6,315		7,754		9,355		6,746		11,304	
Pseudo *R*^2^	0.07		0.06		0.06		0.07		0.06		0.07	
***Indirect effects based on regressions with mediators included***												
Household income	0.043*	0.024	0.040**	0.019	0.032**	0.013	0.046***	0.017	0.058***	0.020	0.019*	0.010
Social functioning	0.117***	0.023	0.037*	0.021	0.057***	0.018	0.119***	0.021	0.102***	0.025	0.076***	0.015
Total indirect effect	0.160***	0.033	0.078***	0.027	0.090***	0.021	0.166***	0.027	0.160***	0.031	0.095***	0.018

Subsample analyses for self-employed individuals in short and long relationships, for self-employed individuals without and with children, and for self-employed individuals living in rural and urban areas. ***p ≤ 0.01, **p ≤ 0.05, *p ≤ 0.10 (two sided). Coeff., coefficient; SE, standard error. The smaller sample sizes are the result of using lagged values to distinguish short from long relationships, and having no children from having children. Urbanization is not known for three individual-year observations. The estimates corresponding to state of residence, sector, and year of the survey are available upon request from the authors.

Important heterogeneity in the impact of separation from the life partner is found based on whether the self-employed individual has employees or not (Table 5). That is, the significant and positive relation between separation and exit from self-employment holds for the self-employed without employees rather than those with employees. Hence, the implications of separation in terms of an exit from self-employment are stronger for self-employed individuals without employees. Furthermore, we find that the significant result for separation applies to both men and women, and that the relationship is significant in the subgroup of lower-educated and younger individuals (with the thresholds set at 12.5 years for education and 45 years for age, the averages in our sample, see Table 1).

In Table 6, we do not find many differences across the subgroups in terms of the relationship between separation from the life partner and the probability of experiencing an exit from self-employment. The significant positive relationship between separation and the probability of an exit from self-employment is found for individuals without and with children living in the household before separation, and also for individuals living in rural and urban areas. However, we find a difference between short-term marriages and *de facto* relationships, and longer-term marriages and *de facto* relationships (threshold set at 16 years, the average duration of marriages and relationships in our sample). The ending of a long-lasting marriage seems to have a stronger positive relationship with exiting self-employment than a short-lasting marriage.

### Robustness Checks

As a first robustness check, we use information about someone’s marital status to construct our “separation variable” rather than the response to the life event question as in our main analysis. The disadvantage of using the marital status question is that information for this variable is needed for two consecutive waves. For example, given that we want to include recent information about separation, it is necessary to use marital status information in both year *t* (i.e., separated) and in year *t* − 1 (for example, married). Hence, the number of separation events in these analyses is lower than in our main analysis (only 229 instances of separation). We repeat our analysis in Table 3 using the marital status variable, and the findings are similar to our original results. That is, there is a significant and positive relationship between separation and the probability of experiencing an exit from self-employment (*b* = 0.51; *p* = 0.001). We find that 12.1% of the relationship is mediated by social functioning (indirect effect is 0.062; *p* < 0.001), and 7.9% is mediated by household income (indirect effect is 0.040; *p* = 0.002). Hence, despite the smaller sample, also in this case, our hypotheses are supported.

The second robustness check entails propensity score matching, where each instance of separation is matched with a non-separation observation in terms of a similar profile for all socio-demographic, job characteristics, and lagged values of social functioning and income. Also here, based on a much smaller sample of 957 observations, the findings support our hypotheses. That is, after our matching procedure, we find a significant and positive relationship between separation and the probability of experiencing an exit from self-employment (*b* = 0.33; *p* = 0.07). In addition, a substantial portion of this relationship is mediated by social functioning (23.5%) and a smaller portion by household income (7.2%).

## Discussion and Conclusion

In this article, it is argued and empirically confirmed that separating from a life partner has significant and far-reaching consequences for self-employed individuals. We found that self-employed individuals are more likely to exit their business after they separated from their life partner. Thus, our results indicate that a separation from a partner not only has consequences within the personal sphere, but also has consequences for the economy through the withdrawal of persons from their businesses. This finding complements and extends prior research that has hinted at the possibility that a separation could lead to an exit from self-employment (Wennberg et al., 2010).

Furthermore, our results suggest that the loss of income and reduced social functioning that may follow a separation partly explain that a separation from the life partner leads to an exit from self-employment. The direct negative consequences of a separation in terms of reduced social functioning and reduced income were already well established. The results of our study suggest that these direct consequences of a separation also may have further far-reaching consequences by impacting exits from self-employment. Although it is well known that one’s financial situation may impact upon the decision to exit from self-employment, our research has disentangled a specific source of income decline (that is, through separating from a partner) that may drive such an exit. Insight into such specific causes of financial deterioration of a self-employed individual prior to an exit not only provides further insight into what causes exits but also gives clues regarding whether responses are needed to deal with such situations to prevent exits, such as through policy making (see below). In addition, our results indicate that the negative social consequences following a separation play a larger role in driving an exit from self-employment than the negative financial consequences. This further supports that exits from self-employment have much broader drivers than only financial ones (Wennberg et al., 2010; Hessels et al., 2018).

We also find that a separation increases the likelihood for a self-employed individual to exit to wage work, i.e., to become an employee within an existing firm. Possibly, without having the social and financial support of a life partner, these individuals will consider self-employment too risky. At the same time, we also find that a separation makes it more likely for the individual who exits self-employment to become self-employed again. This finding is in line with previous studies suggesting that an exit from self-employment often leads to re-entry into self-employment, for example, because one has built up relevant experience and networks for self-employment, or one has a preference for being self-employed (Hessels et al., 2011). Our finding may reflect that after a separation, it may not always be feasible or desirable for the self-employed individual to continue with the current business (e.g., because the partner had an important stake or played an important role in the business) and therefore a new business needs to be created by the individual.

We find that separations are positively related to an exit from self-employment for the self-employed without employees and not for the self-employed with employees. Self-employed individuals with employees experience higher levels of work stress than those without employees (Hessels et al., 2017). They also work under higher pressure (Blanchflower, 2004) and have a higher workload with an additional set of tasks compared to the self-employed without employees (Hébert and Link, 1989; Lazear, 2005). Our results suggest that the continuation of their business is not affected by the separation from a life partner. Possibly, the presence of employees (i.e., social capital) may make a self-employed individual and the functioning of his or her business less dependent on the support of a life partner.

Given the economic impact of exits from self-employment, appropriate responses by policy makers may be warranted. One option could be to provide financial and non-financial aid when self-employed individuals find it difficult to continue with the business after a, possibly expensive, separation from the life partner. Although separation refers to a situation in which two life partners voluntarily decide or arrange to stop living or being together as a couple, (local) governments may, in some cases, want to interfere when effects on welfare are large. In such a case, the government might consider issuing relatively cheap loans for separated self-employed individuals or promote certain social activities more actively among recently separated self-employed. Importantly, policy makers could also consider focusing more on preventing the occurrence of separations, for example, by raising awareness about the potentially serious consequences such as the ones demonstrated in this study.

We have a number of suggestions for future research. First, we would like to encourage researchers to identify other mediators that, next to social functioning and household income, explain the positive association between separating from a life partner and exiting self-employment. Potential mediators may include personality traits that may be affected by a separation from a life partner like (reduced) self-efficacy, or diminished motivation for succeeding with the business. Also, we would like to encourage researchers to gain insight into the motives for exiting self-employment after a separation and to further disentangle to what extent an exit following a separation has been voluntary or not and to what extent such exits are successful (such as a harvest sale) or unsuccessful (such as a forced liquidation) (Wennberg et al., 2010). In addition, researchers could also investigate performance implications for businesses of self-employed individuals who do not exit after a separation. Another suggestion would be to conduct a more in-depth study into the consequences of a separation of individuals who have an equal share in a business. Many business owners have a self-employed spouse (Parker, 2008). Research has focused on why this is the case, but future studies may want to investigate what happens in the event of a separation. It is not only interesting to see which exit routes are followed after experiencing an exit, but also what happens to the business and, whenever applicable, to whom the business is awarded. In a related way, future studies could relate the existing findings to research on family firms. For example, family firms are less likely to exit than non-family firms (Chirico et al., 2019; Madanoglu et al., 2019); one may be interested in what the impact on family firms is when there is a separation event in the family business team.

Finally, our conclusions are based on the analysis of Australian data, and this raises the question as to whether the revealed relationships between separation from the life partner and exit from self-employment are specific to Australia or applicable to other countries as well. In the Introduction, we noted that the demographic patterns in terms of divorce in Australia are similar to trends in other Western countries. However, the prevalence of self-employment is relatively low in Australia. Still, the theoretical considerations that backed up our empirical analyses were not specific for Australia, and therefore, we believe that the relationship between separation from the life partner and exit from self-employment as well as the mediating effects through household income and social functioning are likely to be present in other developed countries as well. However, the strengths of these relationships may differ to some extent across countries, and therefore, future studies may want to validate our findings in other economic contexts.

## Data Availability Statement

The datasets analyzed for this study can be obtained here: https://melbourneinstitute.unimelb.edu.au/hilda/for-data-users.

## Ethics Statement

Ethical review and approval was not required for the study on human participants in accordance with the local legislation and institutional requirements. Written informed consent from the patients/participants/legal guardian/next of kin was not required to participate in this study in accordance with the national legislation and the institutional requirements.

## Author Contributions

LL, JH, CR, and PZ were involved in all parts of the study, except the data analysis. The data analysis was performed by LL and PZ.

## Conflict of Interest

The authors declare that the research was conducted in the absence of any commercial or financial relationships that could be construed as a potential conflict of interest.

## Footnotes

1 Hence, separation does not refer to the death of a life partner.

2 There is one earlier year of data collection but our independent variable was not measured in 2001. The year 2017 was the most recent year of the dataset at the time of writing this paper.

3 Any value between 0 and 1 was transformed into 1 (year), after which the logarithm transformation was applied.

4 The Australian and New Zealand Standard Industrial Classification (ANZSIC) classification was used. Mining was merged with Agriculture, Forestry and Fishing, and Electricity, Gas, Water, and Waste services into Other Services due to relatively few occurrences in these categories.

5 The “number of events” (De Jong et al., 2019) may be an issue in our multinomial logit specification given the relatively few exit occurrences. A robustness check with the so-called penalized likelihood estimator leads to qualitatively similar conclusions.

6 We use 1:1 nearest-neighbor matching with a caliper width of 0.05.

7 We indeed find in supplementary regressions that separation is significantly and negatively related to social functioning (*b* = −12.51; *p* < 0.001) when social functioning is taken as the dependent variable. Hence, while controlling for all other variables in the regression, those who separated experience a drop in social functioning by 12.51 points (on a 0–100 scale) compared with those who do not experience a separation. When the logarithm of household income is taken as the dependent variable, we find *b* = −0.30 (*p* < 0.001).
